# Impact and Outcomes of a Pediatric Robotic Urology Mini-Fellowship

**DOI:** 10.3389/fsurg.2019.00022

**Published:** 2019-04-17

**Authors:** Ciro Andolfi, Dev Patel, Veronica M. Rodriguez, Mohan S. Gundeti

**Affiliations:** Pediatric Urology, Section of Urology, Department of Surgery, The University of Chicago Pritzker School of Medicine, Chicago, IL, United States

**Keywords:** pediatric urology, robotic surgery, robotic-assisted laparoscopy, surgical simulation, surgical training

## Abstract

**Introduction:** In order to support practicing pediatric surgeons and urologists to safely and effectively incorporate robotic surgery into their practice, we established a 5-day mini-fellowship program with a mentor, preceptor and proctor at our institution. This study was designed to report our experience with the pediatric robotic mini-fellowship (PRM) and to evaluate the impact this course had on the participants' practice.

**Methods:** The mini-fellowship training at our institution is provided in two modules, including upper and lower urinary tract surgery, over a 5-day period. The one to one teacher-to-attendee experience included tutorial sessions, hands-on inanimate, and animate skills training, clinical case observations and video discussions. Participants were asked to complete a detailed questionnaire on their practice patterns before and after the PRM.

**Results:** Between 2012 and 2018, a total of 29 national and international pediatric surgeons and urologists underwent robotic renal and bladder surgery training. Twenty-six fellows (90%) completed the surveys, all of which were included for analysis. The median age at the time of fellowship was 43 years (32–63), and participants had practiced urology for a median of 76 months (3–372). All of them had a laparoscopic background, with a median experience of 120 months (12–372), and an average of 454 (± 703) laparoscopic procedures performed, including the years of training. The most common primary goals of participants were to understand the concept of robotic surgery and its applications (38.5%), and to practice in the wet lab to shorten their learning curve (38.5%). After PRM completion, 24 graduates (92%) felt likely to incorporate robotic surgery into their practice, of which 15 (58%) actually started a robotic program at their home institution. At 24 months after PRM completion, the overall number of surgeries performed with a robotic approach (RA) by these 15 participants was 478 with an average of 32 (± 44) procedures per fellow, of which 109 (23%) were extirpative (nephrectomy, partial nephrectomy, etc.), and 369 (77%) reconstructive procedures (pyeloplasty, ureteral reimplantation, etc.). Before PRM, the same 15 participants performed 844 procedures with a laparoscopic approach (LA), of which 527 (62.4%) were extirpative, and 317 (37.6%) were reconstructive surgeries. These data mark a significant switch in indications for minimally invasive surgery (MIS) in pediatric urology. The rise in the number of reconstructive procedures (37.6% LA vs. 77% RA) has shown that robotic surgery has undoubtedly facilitated the performance of more challenging procedures in a minimally invasive fashion.

**Conclusion:** The success of a mini-fellowship program relies on the commitment of expert faculty to serve as tutorial instructors and proctors. In addition, a completely outfitted robotic laboratory with access to dry and wet lab is indispensable. A 5-day intensive PRM appears to enable postgraduate surgeons to successfully incorporate the robotic platform into their practice and to advance the complexity of minimally invasive procedures, allowing for more challenging surgeries, such as reconstructive urology.

## Introduction

After laparoscopy, robotics has revolutionized the minimally invasive approach in surgery. Many advanced and complex procedures have been reported to be feasible and safe by a robotic approach, whereas certain laparoscopic procedures were performed only by remarkably skilled surgeons. As the evidence for clinical safety and advantages of robotic surgery continues to grow, the demand for training will continue to increase. For urology, several groups (1–6) have reported their experience and the impact of structured courses on the participants' practice. However, for pediatric urology such training is not available and this approach has not been thoroughly evaluated so far. In addition, the manufacturers only provide one-day courses, such as the Intuitive Surgical da Vinci training program, which are administered by a technician, without considering human applications, especially for vulnerable populations such as pediatric patients.

At our institution, implementation of a pediatric urology activity with the robotic platform was achieved in October 2007. During our early adaptation, we found that there are a lot of nuances to be learnt for the safe application to the pediatric population. Taking into consideration the increasing success of this technology while also understanding these nuances to reduce the learning curve for colleagues around the world, our division proposed a course in the safe implementation of robotic urology, focused on upper, and lower urinary tract surgery in children. In 2012, we established a 5-day mini-fellowship with a mentor, preceptor and proctor in pediatric robotic urology, to assist practicing pediatric urologists incorporate robotic surgery into their practice. We made this available to those who want to learn as a CME-approved educational and introductory activity. Thus, this study was designed to report our experience with the PRM and to evaluate the impact this course had on the participants' practice.

## Materials and Methods

**Figure 1:**
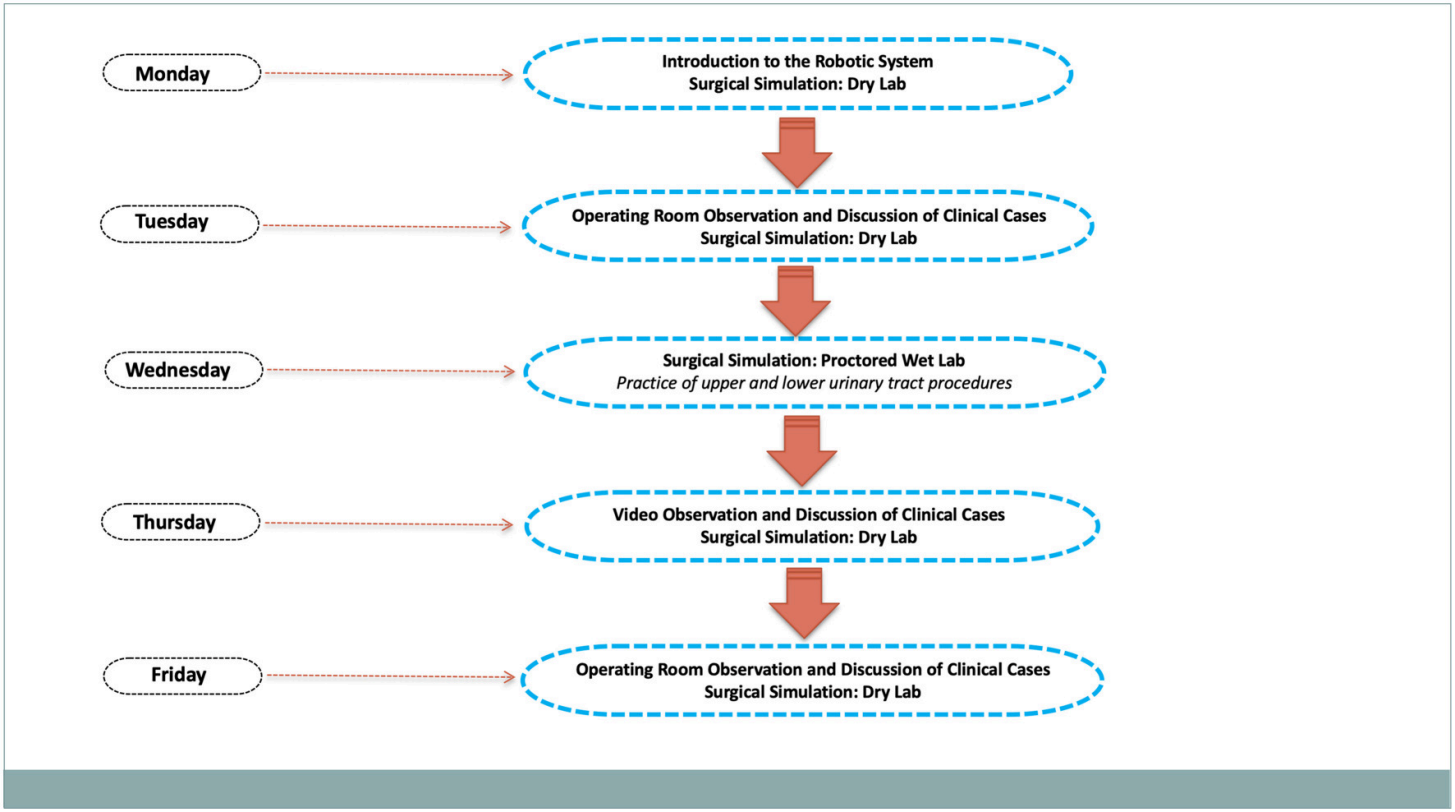
Mini-fellowship schedule flowchart.

The mini-fellowship training at our institution is provided in two modules, including upper and lower urinary tract surgery, over a 5-day period. Participation is reserved to a maximum of three fully-trained pediatric surgeons or urologists, who are asked to go through online modules before training. The PRM embraces a mentor-preceptor-proctor experience and features one to one teacher-to-attendee instruction, including tutorial sessions, hands-on inanimate skills training, animal laboratory practice and operating room observation experience (Figure 1). One fellow would be in the animal lab while another would be in the simulation center doing dry lab, and the third attended the operating room to assist cases. All participants were required to complete a questionnaire before and immediately after completing the PRM to evaluate the various training components using a 5-point Likert scale (from 1—poor to 5—excellent). Program areas assessed were didactic tutorial sessions, skills training practice, animal laboratory training sessions, and operating room observation. As for follow-up, we sent questionnaires to all PRM participants, who were also asked to complete a survey on subsequent robotic operative experience after the PRM program. The study received IRB exemption because it includes interactions involving survey procedures.

All questionnaire results were reviewed, tabulated, and statistically analyzed. Pre-program vs. post-program skill and component ratings were analyzed with a paired sample *t*-test, after logarithmic transformation, and the χ^2^ test was used to analyze data collected on non-continuous variables. A *P* < 0.05 was considered statistically significant.

## Results

Between 2012 and 2018, a total of 29 surgeons, from different Institutions around the world (USA, Canada, Brazil, Mexico, Chile, UK, Italy, Turkey, Saudi Arabia, Lebanon, India, Australia), underwent pediatric robotic renal and bladder surgery training. With a median follow-up of 24 (5–52) months, twenty-six fellows (90%) completed the surveys, all of which were included for analysis. The median age at the time of fellowship was 43 years (32–63), and participants had practiced urology for a median of 76 months (3–372). All of them had a laparoscopic background, with a median experience of 120 months (12–372) and an average of 454 (± 703) laparoscopic procedures performed, including the years of training. The primary goals of participants were to understand the concept of robotic surgery and its applications (38.5%), and to practice in the wet lab to shorten their learning curve (38.5%). After fellowship completion, 24 out of 26 graduates (92%) felt likely to incorporate robotic surgery into their practice, of which 15 (62.5%) actually started their robotic program. Among fellows who did not implement robotic surgery into their practice, financial constraints, and lack of proctorship were the main obstacles. Other impediments were found to be low caseload and absence of simulation training. The vast majority of fellows who implemented robotic surgery into their practice were either academics (60%) or affiliated to an academic institution (33%). The overall number of surgeries performed with a robotic approach (RA) by these 15 PRM participants at 24 months after completion of our program was 478, with an average of 32 (± 44) per fellow, of which 109 (23%) were extirpative (nephrectomy, partial nephrectomy, etc.), and 369 (77%) reconstructive procedures (pyeloplasty, ureteral reimplantation, etc.). Before PRM, the same 15 participants performed a total of 844 procedures with a laparoscopic approach (LA), of which 527 (62.4%) were extirpative and 317 (37.6%) reconstructive surgeries. These data mark a significant switch in indication and use of minimally invasive surgery (MIS) in pediatric urology, with a rise of reconstructive procedures when a robotic platform is available (37.6% LA vs. 77% RA, *P*<*0.01*). The number of high-grade complications reported was 14 (2.9%), all Clavien-Dindo grade III. The average number of operations needed to reach a subjective level of confidence was inversely proportional to the age of patients — from 4 for 5-year old patients to 10 for patients younger than 2 years of age. Seven (47%) out of 15 reported an independent level of performance, 3 (20%) required supervision, while the remaining 5 (33%) were able to perform only part of the procedure. In order of importance, the most anxiety-provoking components of surgery were: (1) suturing and tying; (2) accidental injury to surrounding structures; (3) ports positioning and handling; and (4) dissection. Overall, the PRM received an evaluation score of 4.5 (± 0.6) and, when fellows were questioned if they would recommend the PRM to other colleagues, the average score on a Likert scale was 4.7 (± 0.5). The two most useful elements of the PRM were found to be (1) wet lab training and (2) operative room observation. Among fellows who were able to embrace the robotic technology after the PRM a subjective improvement of the following skills was noted when compared to laparoscopy: ports management (3.9 vs. 4.5, *P* = *0.045*); dissection (3.9 vs. 4.5, *P* = *0.032*); clipping and stapling (3.8 vs. 4.5, *P* = *0.019*); cutting tissue (3.9 vs. 4.6, *P* = *0.025*); suturing and tying (3.1 vs. 4.4, *P* = *0.002*).

## Discussion

Robotic surgery is increasingly gaining support from the surgical community. There has been a call from major governing bodies such as the Society of Urologic Robotic Surgeons, European Association of Urology, and the American Urological Association for development of training curricula increasing preclinical exposure to surgical techniques and validated assessment tools of proficiency (7). Several projects are currently underway to develop a robotic surgery curriculum and are in various stages of validation. Whilst the Fundamental Skills of Robotic Surgery (FSRS) is based on tasks on the Robotic Surgical Simulator (RoSS) virtual reality simulator; Fundamentals of Robotic Surgery (FRS) and Basic Skills Training Curriculum (BSTC) are designed for use on simulators and the robot itself (8–10). The European Association of Urology (EAU) Robotic Urology Section (ERUS) curricula benefits from a combination of dry lab, wet lab and virtual reality components, which may allow skills to be more transferable to the OR as tasks are completed in several formats. Finally, the ERUS curriculum includes the OR modular training program, as table assistant and console surgeon, and a post-training mentorship system which allows the trainees to assess their proficiency (11). However, the curriculum is in early stages of validation and more work is needed. In addition, there is no specific module tailored to pediatric patients.

Because of these limitations, pediatric surgeons have become increasingly eager to learn and adopt robotic techniques for the pediatric population despite having had little or no training during residency. Traditionally, for individuals not extensively exposed to robotic surgery during residency, robotic skills are learned by pursuing a post-residency fellowship in MIS or during brief one-day courses. For practicing urologists who seek training in robotic surgery, these short courses may not instill enough confidence or the necessary skill set to perform these procedures independently. At the same time the additional rigors of academic training imposed by dedicated fellowships preclude most urologists from obtaining appropriate training. To achieve better results than short courses in terms of take rates, while curtailing the time required for a formal MIS fellowship, we developed a dedicated five-day PRM program at our institution. As in the ERUS curriculum, we provided a non-formal post-training mentorship in order to help former fellows implement the robotic system into their daily routine. This is done remotely, through online networking and workshops, and at their home institution, through on-site training sessions, when feasible. The formalized implementation of such endeavors will need additional resources.

For this study, PRM participants were contacted 2 years after training and responded to a questionnaire specifically looking at practice patterns. Even if the vast majority of participants felt likely to incorporate robotic surgery into their practice, slightly over half of them had actually done so. Identifying ways to improve the take rate for robotic surgery is an important element in permitting PRM graduates to use these skills. Based on our data, financial constraints and lack of proctorship at the home institution were the biggest barrier for those who did not implement robotic surgery into their practice. Hopefully, the prohibitive costs of currently available robotic platforms will drop with the market inflow of new robotic technologies. However, proctorship will still be a challenge as, in the absence of a uniform training, credentialing and privileging remain institution-based processes.

After many years of exciting progress and refinements of laparoscopic instrumentations, the application of conventional MIS to the pediatric population has reached a plateau, particularly for reconstructive procedures. Intracorporeal suturing using the available laparoscopic tools is time consuming and requires vast experience. The robot, instead, has the potential to overcome these limitations by providing enhanced depth perception and improved dexterity with fine motions skills, and reducing the learning curve of intracorporeal tasks, such as dissection and suturing. As a result, surgeons are adopting this technology to assist in surgical procedures that were not feasible with conventional MIS. This observation is strongly supported by our study results, which show how our PRM program has dramatically changed indications to use an MIS approach. Among the 15 participants who established a robotic program in their home institutions, the rate of reconstructive procedures performed with an MIS approach, rose from 37.6 with laparoscopy to 77% with robot-assisted surgery. In addition, these fellows reported a subjective improvement with the robot in surgical skills, such as dissection, cutting, suturing, and tying.

To our knowledge the PRM in robotic pediatric urology remains unique to our institution. While regrettable, it is understandable, given the considerable commitment in personnel, space, equipment, and funds needed to equip and maintain a training facility of this nature. Because we value sharing skills that will unquestionably promote children's safety, we made a commitment to pursue this program to help educate practicing physicians on the benefits and usages of robotic surgical techniques. The success of a PRM program relies on the commitment of expert faculty to serve as tutorial instructors and proctors. In addition, a completely outfitted robotic laboratory with access to dry and wet lab is indispensable. A 5-day intensive robotic pediatric urology PRM appears to enable postgraduate surgeons to successfully increase their case volume and advance the complexity of minimally invasive procedures.

## Author Contributions

CA and MG: design of the work. CA, DP, VR, and MG: drafting the work and revising it critically. CA, DP, VR, and MG: final approval. All authors agree to all aspects of the work.

### Conflict of Interest Statement

MG is co-director for the NARUS course. The remaining authors declare that the research was conducted in the absence of any commercial or financial relationships that could be construed as a potential conflict of interest.
